# Eosinophil counts as a relevant prognostic marker for response to nivolumab in the management of renal cell carcinoma: a retrospective study

**DOI:** 10.1002/cam4.4208

**Published:** 2021-08-18

**Authors:** Tressie Herrmann, Angeline Ginzac, Ioana Molnar, Sébastien Bailly, Xavier Durando, Hakim Mahammedi

**Affiliations:** ^1^ Département d’Oncologie Médicale Centre Jean PERRIN Clermont‐Ferrand France; ^2^ Université Clermont Auvergne UFR Médecine Clermont‐Ferrand France; ^3^ INSERM U1240 Imgerie Moléculaire et Stratégies Théranostiques Université Clermont Auvergne Centre Jean PERRIN Clermont‐Ferrand France; ^4^ Centre d'Investigation Clinique UMR 501 Clermont‐Ferrand France; ^5^ Division de Recherche Clinique Délégation Recherche Clinique et Innovation Centre Jean PERRIN Clermont‐Ferrand France; ^6^ Département d’Oncologie Médicale CHU Gabriel Montpied Clermont‐Ferrand

**Keywords:** Biomarker, Eosinophils, nivolumab, Renal cell carcinoma

## Abstract

**Background:**

Despite improvements in the management of renal cell carcinomas (RCC) with the advent of immunotherapy, only a few patients respond to these treatments. Predictors of response to nivolumab are currently being investigated but are still lacking.

**Aim of the study:**

To evaluate eosinophil levels and their variations during treatment as an accurate biomarker for outcome in metastatic RCC treated with nivolumab.

**Methods:**

A retrospective analysis was carried out for patients with metastatic RCC treated with nivolumab. Absolute eosinophil counts, their variation, and relative change were evaluated at six weeks. Relative eosinophil change was categorized in three groups (≥10%‐decrease, no change, ≥10%‐increase). Univariable and multivariable analyses were performed to determine whether eosinophils and their variations were prognostic markers for response at the first scan evaluation, progression‐free survival, and overall survival.

**Results:**

Sixty‐five patients aged on average 66 years, 68% men, and 77% with good or intermediate International Metastatic Renal Cell Carcinoma Database Consortium (IMDC) risk group were included. The median follow‐up was 16.6 months. Median overall survival (OS) was not reached for good prognosis and was 22.5 and 6.5 months for intermediate and poor prognosis, respectively. An increase in eosinophils and relative eosinophil change at six weeks of nivolumab was associated with a good response to immunotherapy (*p* = 0.012 and *p* = 0.024 respectively). In the group of patients with a 10%‐decrease in relative change, PFS reduced significantly compared to the other groups (*p* = 0.0044 with the 10%‐increase group and *p* = 0.03 with the no‐change group). This relative increase was independent of IMDC risks factors for better OS (HR = 3.3 [1.45–7.4]; *p* = 0.004). The eosinophil baseline level was not associated with response to treatment.

**Conclusion:**

Eosinophil levels and relative eosinophil change at 6 weeks might be good prognostic markers for response to nivolumab for metastatic RCC, and were associated with better PFS and OS.

## Graphical abstract

Despite improvements in the management of renal cell carcinomas (RCC) with the advent of immunotherapy, only a few patients respond to these treatments. Predictors of response to nivolumab are currently being investigated but are still lacking. Eosinophil levels and relative eosinophil change at 6 weeks might be good prognostic markers for response to nivolumab for metastatic RCC and were associated with better PFS and OS.

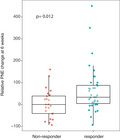

## 1 INTRODUCTION

Renal cell carcinoma (RCC) is a rare disease accounting for 3%–5% of all malignancies. In 2018, about 330 000 new cases have been diagnosed around the world, with an increasing incidence especially in developed countries.^1^, ^2^ This increase was due to an aging population and also to improvements in imagery technologies.^3^, ^4^ Because of a lack of clinical symptom, RCC is detected at a metastatic stage in 30%–50% of cases and is associated with a poor prognosis, with a 5‐year median survival of 10%.

RCC is not chemo‐sensitive and was characterized as a radio‐resistant tumor before the advent of stereotactic body radiation therapy.^5^, ^6^ Treatment has for a long time been solely based on surgical strategies. Current guidelines are now recommending targeted therapies with less toxicity and higher survival benefits. They have become the mainstay of treatment for metastatic RCC (mRCC), and multiple targeted therapies, such as tyrosine kinase inhibitors (TKI), mammalian target of rapamycin pathway inhibitors (mTOR), and vascular endothelial growth factor (VEGF) monoclonal antibody, have all been approved as first‐line systemic treatments for mRCC. More recently, immune checkpoint inhibitors (ICI) have been promoted as a further therapeutic option.

Nivolumab is an anti‐programmed death 1 (PD1) IgG4 antibody which was the first checkpoint inhibitor to be approved for the management of metastatic RCC refractory to other targeted therapies in November 2015.^7^ The CheckMate‐025 trial has demonstrated that nivolumab improved OS in comparison with everolimus.^8^, ^9^, ^10^ Tolerance was good with only 19% grade 3 or 4 adverse events versus 37% in the everolimus group. In 2017, the CheckMate‐214 trial has demonstrated better OS and objective response rates (ORR) with a combination of nivolumab plus ipilimumab, rather than sunitinib, among patients with intermediate and poor prognosis according to the International Metastatic Renal Cell Carcinoma Database Consortium (IMDC) risk's factors in the first line of metastatic RCC.^11^, ^12^ Other studies have evaluated treatment combinations, such as nivolumab plus cabozantinib (CheckMate 9ER),^13^ or pembrolizumab plus axitinib (KEYNOTE‐426)^14^, ^15^ for metastatic RCC. The two above‐mentioned trials highlighted improvements in terms of progression‐free survival (PFS) and OS for nivolumab plus cabozantinib; and for pembrolizumab plus axitinib. These associations have been standard of care for first‐line metastatic RCC since 2019.^16^

The current evolution in the management of RCC is very encouraging, but only a few patients actually benefit from immunotherapy.^9^, ^13^, ^15^, ^17^ Current research aims to find reliable predictive clinical or biological markers to predict response to nivolumab, but so far little has been achieved. Indeed, as shown in the CheckMate 025 trial, both groups with high or low PD‐ligand 1 (PD‐L1) expression benefited from immune treatment. This revealed that PD‐L1 expression was prognostic but not predictive of response to nivolumab. On‐going studies are being carried out to find and select predictive markers, such as tumor mutational burden,^18^, ^19^ tumor‐infiltrating lymphocytes^20^ or gene expression signatures^21^, ^22^, ^23^, ^24^, ^25^ but due to the complexity of clinical routines, serum markers are readily available for the analysis are currently being investigated. One study conducted recently suggested a rise in eosinophils as a predictive biomarker for ORR, OS, and PFS in metastatic RCC treated with nivolumab.^26^

The prognostic role of eosinophils is still controversial, because of discordant results depending on the types of carcinoma.^27^, ^28^, ^29^, ^30^, ^31^ Patients with gastrointestinal cancer, non‐small cell lung carcinoma, or metastatic melanoma had better survival with eosinophilia while patients with Hodgkin's lymphoma had poorer outcomes. A few studies carried out on small cohorts of patients with melanoma or lung carcinoma treated with immunotherapy proved that monitoring eosinophil counts or variations could be predictive of patient response.^32^, ^33^

The main objective of our trial is to investigate the impact of eosinophils, and their early variation at 6 weeks under nivolumab for outcomes among patients with metastatic RCC.

## 2 PATIENTS AND METHODS

### 2.1 Population

A retrospective study was conducted on patients treated with Nivolumab for mRCC in two centers in Clermont‐Ferrand from 2016 to 2021. A total of 65 patients were included. Each patient has been informed about the research by a non‐opposition letter. They were free to oppose to the use of their personal medical data.

According to French legislation, the database was notified to the CNIL (the French regulatory body for data privacy). Ethics approval for the study was obtained on 26 April 2021 (« *Comité d’Ethique des Centres d’Investigation Clinique»* (CECIC) Rhône‐Alpes‐Auvergne, Grenoble, IRB 5921).

### 2.2 Data collected

All clinical information was recovered from the patients’ electronic medical records. The following patient medical data were collected: past history of nephrectomy; Fuhrman's nuclear grade; histology; IMDC risks factors (such as hemoglobin, platelets, absolute neutrophil count, corrected calcium, Eastern Cooperative Oncology Group Performance Status (ECOG PS), and time from diagnosis to systemic treatment); number of previous systemic treatments; location of metastases; and eosinophil levels at the initiation of nivolumab, at 6 weeks and at the time of the first evaluation; duration of immunotherapy; results at first evaluation; scan date of progression; toxicities; date of death or last follow‐up assessment.

### 2.3 Patient follow‐up

The first evaluation was either conducted at 6 weeks or after approximately 12 weeks of treatment, corresponding to 3 or 6 immunotherapy injections respectively. Progression, response or disease stability were evaluated using the Response Evaluation Criteria in Solid Tumor (*version irRECIST*) by an expert radiologist.

### 2.4 Statistical analysis

Statistical analyses were carried out using the R environment (https://cran.r‐project.org). Categorical variables were presented as counts and percentages. Quantitative variables were summarized as medians and ranges. We investigated the impact of baseline eosinophil counts on the first evaluation results. The responder group was defined as complete response, partial response or stability at the first evaluation, while the non‐responders were defined as exhibiting hyper‐progression or progression.

Progression‐free survival was calculated as the time from the first nivolumab injection to radiographic or clinical progression or death (whichever came first). OS was evaluated as the time from the first nivolumab injection to the date of death or last follow‐up. The Kaplan–Meier curves were compared using the log‐rang test (using the R “survival” package). Multivariable analysis was performed using the Cox proportional hazard regression model.

The relative change in eosinophils was calculated as follows: ([eosinophils week 6/eosinophils day 0] –1)*100 and categorized into three groups (≥10% decrease, no change [<10% decrease to <10% increase], ≥10% increase). We analyzed the correlation between the relative change in eosinophils and the results at primary evaluation, PFS, and OS.

Statistical tests used are in agreement with data distribution: normality was first checked using the Shapiro–Wilk test and parametric (Student's t test) or non‐parametric (Mann–Whitney test) two‐tailed test was applied according to normality respect. Comparisons between two categorical variables were performed using Fisher's Exact Test.

Differences were considered to be statistically significant at values of *p* < 0.05.

## 3 RESULTS

### 3.1 Baseline characteristics and outcomes

Sixty‐five patients were included in this study. The baseline characteristics are described in Table 1. The median age at the first dose of nivolumab was 66 years (range 37–86) and there was a majority of men (68% vs. 32%). Clear cell renal carcinoma (CCRC) was the most frequent histology with 89%, versus 11% for non‐CCRC (papillary tumors or chromophobe renal cell carcinoma). Twenty‐two percent of the population had a favorable risk, 55% an intermediate risk, and 23% a poor IMDC risk. Prior nephrectomy had been carried out for 48 patients and most patients had received one prior systemic therapy. The most common sites of metastases were the lungs and lymph nodes, but many patients had several concomitant sites of metastases. Patients were ECOG PS 0 or 1 at baseline: only one was ECOG PS 3 at the beginning of the immune therapy.

**TABLE 1:** Baseline patient characteristics.

		Number	Percentage
Median age, years		66 (37–86)	
Gender	Female	21	32
	Male	44	68
Past of nephrectomy	Yes	48	74
	No	17	26
Histology	Clear Cell Renal Carcinoma	58	89
	Non‐clear Cell Renal Carcinoma	7	11
Fuhrman's Nuclear Grade	NA	17	26
	1	2	3
	2	20	31
	3	17	26
	4	9	14
Number of prior systemic therapies	0	1	2
	1	43	66
	2	15	23
	≥ 3	6	9
IMDC Risk Group	Favorable	14	22
	Intermediate	36	55
	Poor	15	23
Sites of metastases at baseline	Lung	47	
	Liver	14	
	Bone	22	
	Lymph Node	37	
	Brain	9	
	Pancreas	9	
	Adrenal	7	
	Others	12	
ECOG PS at baseline	0	22	34
	1	28	43
	2	14	22
	3	1	2

The median follow‐up was 16.6 months. According to IMDC risks factors, median PFS was 20.7 months for good prognosis, 6 and 2.4 months for intermediate and poor prognosis respectively. Median OS was not reached for good prognosis, indeed, only four patients among 14 died at the end of the follow‐up, which represents 71.4% of overall survival. Median OS was found to be 22.5 and 6.5 months for intermediate and poor prognosis respectively (supplementary figure). At the end of the analysis, 13 patients were still being treated with nivolumab and six had ceased this treatment because of toxicities.

### 3.2 Absolute value of eosinophils

The median level of eosinophils at day 1 of nivolumab was 0.13 G/L (min = 0.02; max = 0.39). No statistical difference between the IMDC groups and outcome was observed, nor for the other evaluation time points (6 weeks and 3 months).

### 3.3 Variations in eosinophil counts during treatment and results of the first evaluation

**FIGURE 1:**
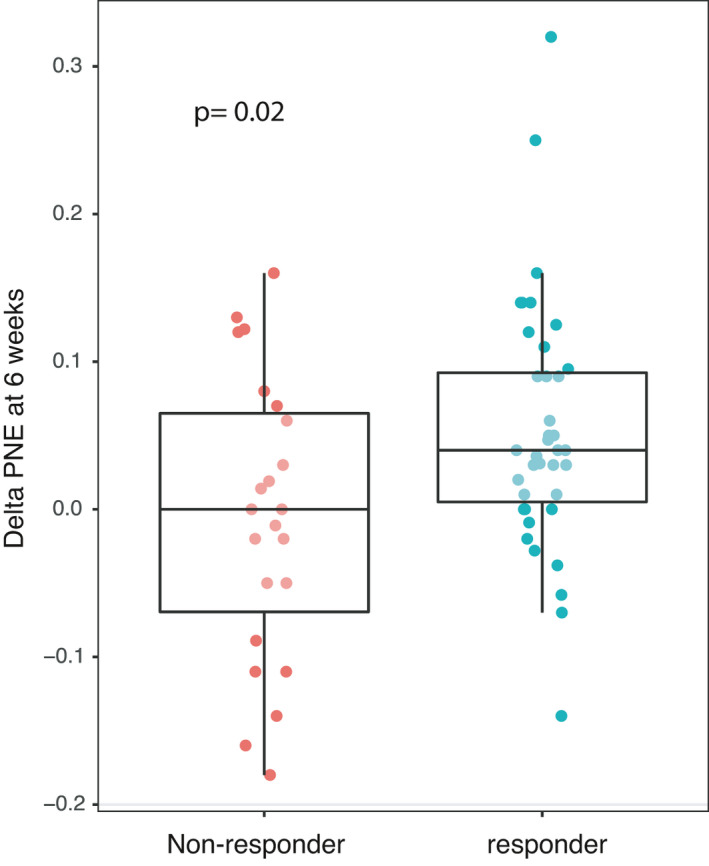
Delta of the polynuclear eosinophils (PNE) according to response to nivolumab at 6 weeks.

The variation in eosinophil counts (noted as delta) was defined as the absolute value for eosinophils at 6 weeks minus the value on the day of the first nivolumab injection. This delta was correlated with scan results at the first evaluation (*p* = 0.02; Mann–Whitney test) (Figure 1).

### 3.4 Predictive role of relative eosinophil change at 6 weeks

**FIGURE 2:**
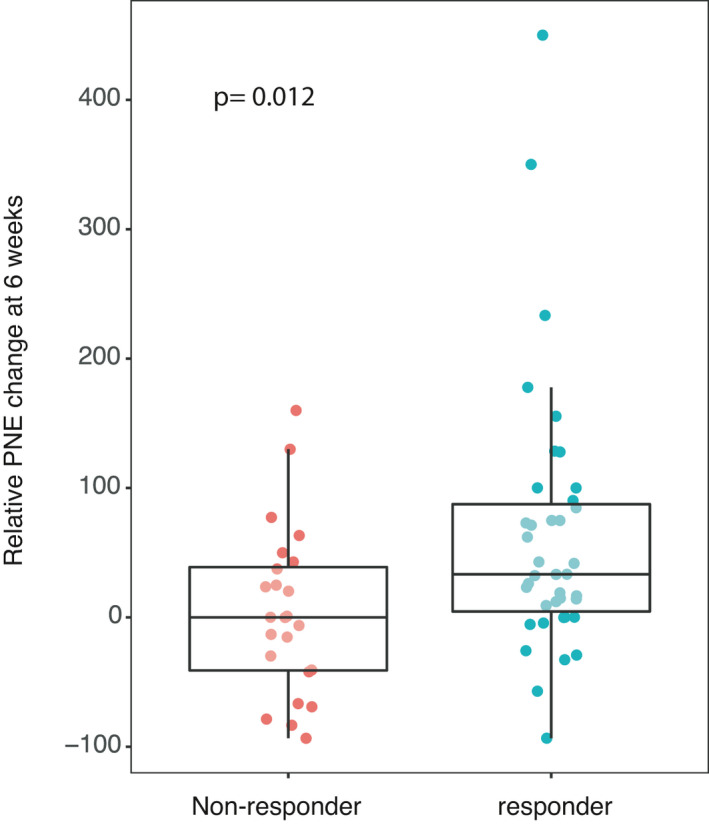
Relative polynuclear eosinophil change at 6 weeks according to response to nivolumab.

Relative eosinophil change as previously described was a good predictor of results at first evaluation (Figure 2). An increase in this value predicted response at first evaluation (*p* = 0.012; Mann–Whitney test). An increase of 10% in this relative change was a good predictive factor of the results at primary evaluation (*p* = 0.024; Fisher's Exact Test).

### 3.5 Prognostic role of relative eosinophil change at 6 weeks

**FIGURE 3:**
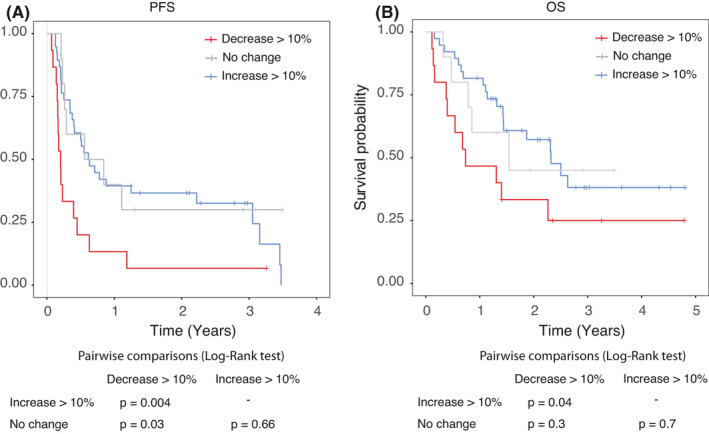
PFS (a) and OS (b) according to the 3 groups of relative eosinophil change, i.e. ≥10% increase; no change and ≥10% decrease respectively.

Relative eosinophil change was categorized into three groups (≥10% decrease, no change [<10% decrease to <10% increase], ≥10% increase. There were 15, 10, and 38 patients respectively in the different groups. Relative eosinophil change from the baseline of treatment with nivolumab predicted PFS and OS. There was no statistical difference with “no change” group and groups with variation in terms of OS. In the group of patients with a 10 percent decrease, PFS reduced significantly compared to the other two groups (*p* = 0.0044 for 10 percent increase and *p* = 0.03 in the no change group; Log‐Rank test) (Figure 3a,b).

**FIGURE 4:**
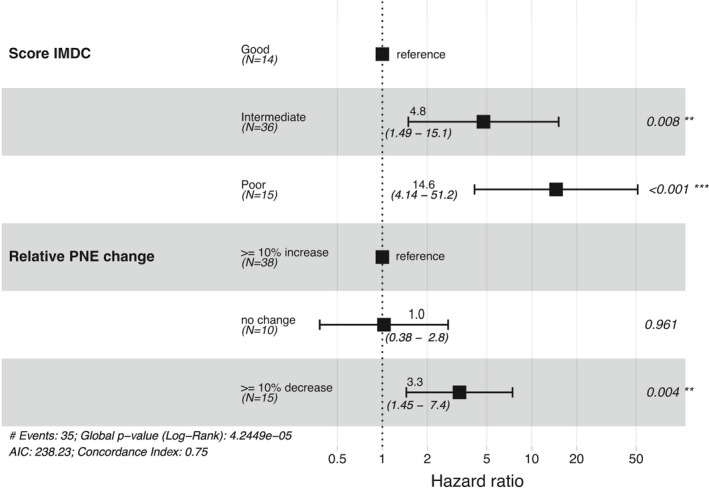
OS in the multivariable analysis according to IMDC risks factors and the relative change in eosinophil counts during treatment.

In Cox univariable analysis, a 10 percent decrease was independently associated with poorer OS (HR =2.15 [1.02–4.5]; *p* = 0.04, data not shown). When the IMDC variable is added in the Cox multivariable model, relative eosinophil change appeared to be independently associated with an improvement in OS. There was no statistical difference between the no change group and the group with an increase in relative change. Variations between ten percent decrease and the increase appeared to be correlated respectively with a decrease or increase in OS (HR =3.3 [1.45–7.4]; *p* = 0.004) (Figure 4).

The multivariable model included two factors: score IDMC and relative PNE change, because they were statistically significant in univariable analysis. Indeed, demographic parameters such as sex, BMI, and age were tested but appeared to be not significant (data not shown).

## 4 DISCUSSION

The main objective was to evaluate the impact of the variation of eosinophil counts on outcomes for patients treated with nivolumab for metastatic RCC. In this study, patient characteristics were comparable to those reported in most recent studies carried out on metastatic RCC, especially for histology, IMDC risks factors, duration of immune therapy, PFS, and OS. We showed that an early increase in eosinophils was associated with a good response to treatment. A 10% increase in the relative change at 6 weeks was predictive of the response at first imagery evaluation, while the 10 percent decrease was correlated with progression. Significant improvement in OS and PFS was observed with the relative change in eosinophil levels under immune checkpoint inhibitors (ICI). Relative change, independent from IMDC risks factors, was also associated with better OS for patients treated with nivolumab for metastatic RCC. However, we did not observe any significant association between eosinophil counts at baseline or quantitative values at primary evaluation and response to treatment.

Due to its retrospective nature and the low frequency of kidney carcinomas, this study presents some limitations. Only 65 patients treated with nivolumab in the two selected center during the given period were included, and information on nuclear grade were missing for 26% of the cases. However, a strength of our study is to assess a simple and cost‐effective marker that is available in every blood sample and can be monitored routinely. Furthermore, to our knowledge, few studies have focused on this subject. Our trial suggests that the relative change in eosinophils at 6 weeks is a prognostic marker for first scan results, PFS and OS in metastatic RCC treated with nivolumab, while this is not the case for baseline values.

Our data have led us to wonder whether tumor control could be mediated by the induction of eosinophils when nivolumab is initiated among patients with metastatic RCC. It has been shown that eosinophils could be cytotoxic against tumor cells, even though their role is still controversial in the literature. In the work by Carretero et al., using an anti‐eosinophil agent (siglec‐F‐specific antibody) in mice models, the authors proved that a decline in eosinophils induced tumoral growth and shorter OS in their mice. Their work suggested that eosinophils play an active part in an antitumoral action, through CD8^+^ T cells but not directly.^34^ Eosinophils could participate in antitumorigenic activity by secreting chemoattractants such as CC‐chemokine ligand 5 (CCCL5), Chemokine (C‐X‐C motif) ligand 9 (CXCL9), and CXCL1. The production of such chemokines could enable CD8^+^ T cells to be attracted to and activated in the tumor.^34^, ^35^ Eosinophils also shape the tumor microenvironment by regulating the vascular system, among other properties, and might display either a direct or an indirect role in tumoral rejection.^30^, ^36^ In fact, eosinophil infiltration into tumors and peripheral eosinophil blood levels were found to be predictive of good or bad prognosis depending on the type of tumor.^31^ Because of their implication in anti‐tumor response, eosinophil counts and their changes have been investigated. In melanomas, monitoring of the evolution of eosinophils during immune treatment was associated with better OS.^32^, ^37^, ^38^, ^39^ Similar results were observed in NSCLC. In multivariable analysis, a significant positive correlation between improved OS and a rise in eosinophils was observed.^33^, ^40^ In RCC, Wang et al. showed that eosinophils could be a good predictive marker, because patients who had elevated eosinophil counts responded better to sorafenib.^41^ More recently, in a Dutch retrospective multicenter trial, researchers demonstrated that an increase in eosinophils at 8 weeks can be used as a biomarker for ORR, PFS, and OS for patients receiving nivolumab.^26^ In this research, other biomarkers were discussed, such as LDH or lymphocytes, which were correlated with OS, PFS, and ORR. The demonstration of the prognostic role of these lymphocytes corroborated Lalani et al.*’s* findings.^42^ Indeed, they used the early relative change in NLR at 6 weeks as a marker for response to ICI for metastatic RCC and showed that it was independently correlated with OS, and PFS. In our cohort, there was a correlation between relative NLR change and PFS but not with OS (data not shown).

Approved markers in NSCLC or melanoma, such as levels of PDL‐1 expression or TMB, have failed to discriminate good from poor metastatic RCC responders to immunotherapy.^25^, ^43^ In the CheckMate 025 trial, the two groups with high or low PD‐L1 expression benefited from immune treatment.^10^ Increasing the expression cut‐off for PDL‐1 expression did not impact ORR in the co‐administration of pembrolizumab and axitinib.^14^ The results demonstrated that PD‐L1 expression was prognostic but not predictive for response to immunotherapy.

Because of the significant formation of neoantigens on the tumor surface, TMB is considered a good predictive factor for response to immune treatment.^19^ Genomic profiling of a large variety of solid tumors was performed, and with a cut‐off at 20 percent, the authors emphasized an improvement in OS across the whole cohort.^44^ Nevertheless, several trials have demonstrated that TMB does not reliably predict response in RCC.^44^, ^45^, ^46^ Gene expression profiling using RNA‐sequencing has enabled the definition of various subtypes of RCC providing a certain degree of immune involvement and angiogenesis. In retrospective studies, these signatures may be correlated with better ORR, PFS, and OS.^47^, ^48^, ^49^, ^50^ In a recent review, Pourmir et al. detailed the main current predictive biomarkers assessed in kidney carcinomas, focusing on genomic signatures.^51^ The BIONIKK trial highlighted the tumor molecular characteristics for the selection of the appropriate treatment among several options currently available for patients suffering from metastatic RCC.^23^, ^24^ These observations are encouraging lines of research to predict outcomes in this rare disease, and they highlight the urgent need to select accurate predictive markers. Nevertheless, readily available markers such as eosinophils are required to adapt treatment in the new landscape of personalized medicine in routine clinical practice.

Further prospective studies are required to consolidate our findings. For instance, eosinophils and their variation should be assessed for combinations of treatments, particularly pembrolizumab plus axitinib, cabozantinib plus nivolumab, which are the current reference, or lenvatinib plus pembrolizumab, which is a novel approach to the management of advanced metastatic RCC.^17^

## 5 DATA AVAILABILITY STATEMENT

The data that support the findings of this study are available from the corresponding author upon reasonable request.

## CONFLICT OF INTEREST

No potential conflict of interest was disclosed by the authors.

## AUTHOR CONTRIBUTIONS

TH recruited patients, interpreted data, wrote and revised the manuscript. SB participated in the patient recruitment and revised the manuscript. IM analyzed the data. HM designed the study, revised the paper, and supervised the project. AG critically revised the manuscript. XD critically revised the manuscript. All authors read and approved the final manuscript.

## ETHICAL APPROVAL

Study ethics approval was obtained on 26 April 2021 (CECIC Rhône‐Alpes‐Auvergne, Grenoble, IRB 5921).

## Supporting information

Fig S1
