# Supplementary figures and images for: Eosinophil counts as a relevant prognostic marker for response to nivolumab in the management of renal cell carcinoma: a retrospective study
Source: Cancer Med. 2021 Aug 18;10(19):6705–13. doi: 10.1002/cam4.4208 (PMC8495279; doi:10.1002/cam4.4208)

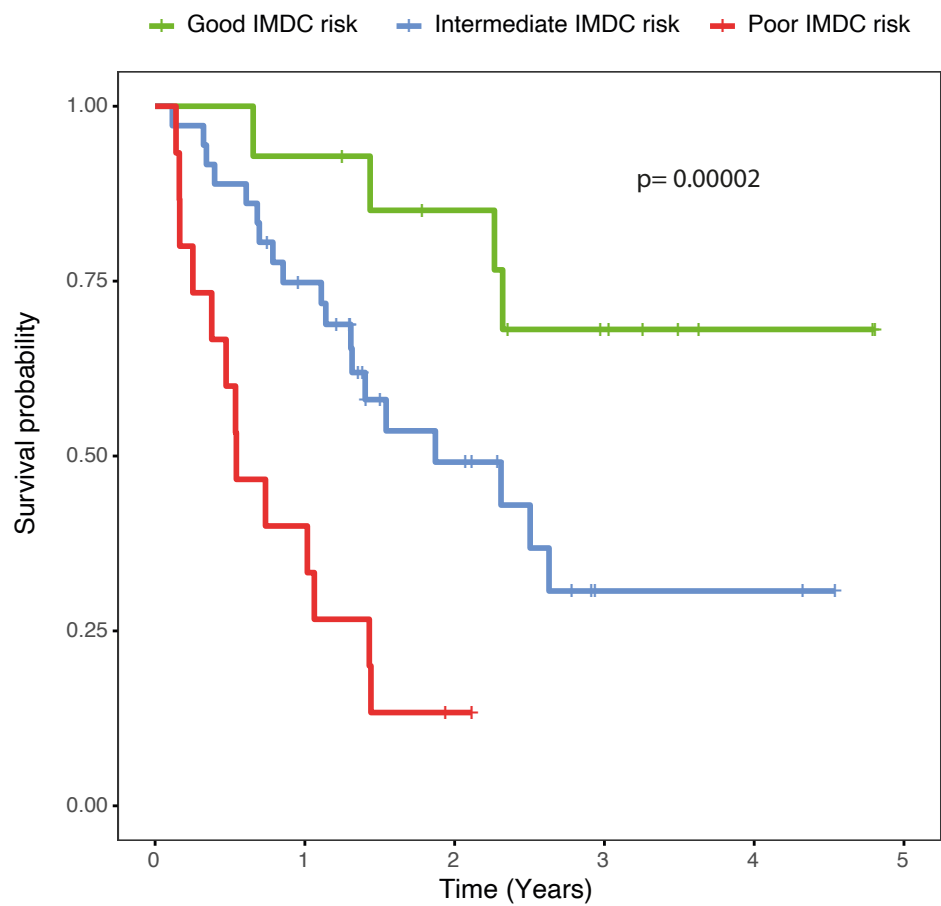

Supplement: Supplementary file 1 — Fig S1 [file CAM4-10-6705-s001.pdf]
